# A GAL80 Collection To Inhibit GAL4 Transgenes in *Drosophila* Olfactory Sensory Neurons

**DOI:** 10.1534/g3.118.200569

**Published:** 2018-09-27

**Authors:** Jessica Eliason, Ali Afify, Christopher Potter, lchiro Matsumura

**Affiliations:** * Janelia Research Campus, Howard Hughes Medical Institute’ Ashburn, VA, 20147; † Department of Biochemistry; Emory University School of Medicine; Atlanta, GA, 30322; ‡ Solomon H. Snyder Department of Neuroscience; Johns Hopkins University School of Medicine; Baltimore, MD, 21205

**Keywords:** Olfaction, GAL80, Drosophila, Odorant Receptor, Sensory Neurons

## Abstract

Fruit flies recognize hundreds of ecologically relevant odors and respond appropriately to them. The complexity, redundancy and interconnectedness of the olfactory machinery complicate efforts to pinpoint the functional contributions of any component neuron or receptor to behavior. Some contributions can only be elucidated in flies that carry multiple mutations and transgenes, but the production of such flies is currently labor-intensive and time-consuming. Here, we describe a set of transgenic flies that express the *Saccharomyces cerevisiae* GAL80 in specific olfactory sensory neurons (*OrX-GAL80s*). The GAL80s effectively and specifically subtract the activities of GAL4-driven transgenes that impart anatomical and physiological phenotypes. *OrX-GAL80s* can allow researchers to efficiently activate only one or a few types of functional neurons in an otherwise nonfunctional olfactory background. Such experiments will improve our understanding of the mechanistic connections between odorant inputs and behavioral outputs at the resolution of only a few functional neurons.

The olfactory system of *Drosophila melanogaster* is often the subject in studies of memory, evolution, gene choice, development and odorant-induced behavior. It is a good model system because of its relatively stereotyped neuronal circuitry, complex behaviors and convenient genetic tools.

In *Drosophila*, most olfactory sensory neurons (OSNs) typically expresses a single odorant receptor (OR) from a genomic repertoire of 60 genes (Vosshall *et al.* 1999; Robertson, Warr, and Carlson 2003; Vosshall, Wong, and Axel 2000; Clyne *et al.* 1999; Goldman *et al.* 2005). The promoter of an OR gene can be employed to label specific subsets of OSNs with a particular transgene (Fishilevich and Vosshall 2005; Couto, Alenius, and Dickson 2005). ORs, which vary in sensitivity and specificity to a wide range of different odorants, determine the firing kinetics and odor-response dynamics of each OSN (Hallem and Carlson 2006; Hallem, Ho, and Carlson 2004; Couto, Alenius, and Dickson 2005; Fishilevich and Vosshall 2005; de Bruyne, Clyne, and Carlson 1999; de Bruyne, Foster, and Carlson 2001; Dobritsa *et al.* 2003; Elmore *et al.* 2003; Kreher *et al.* 2008).

Most OSNs express Odorant Receptor Co-Receptor (Orco), a highly conserved member of the olfactory receptor family (Krieger *et al.* 2003; Vosshall and Hansson 2011), in addition to a single selected OR. Though Orco usually does not contribute to the structure of the odorant binding site (Jung, borst, and Haag 2011; Nakagawa and Vosshall 2009; Nichols and Luetje 2010; P. L. Jones, Pask, and Rinker 2011), it is essential for odorant-invoked signaling in flies. Without Orco, the co-expressed OR cannot localize to the dendritic membrane or relay an odor-evoked signal (Larsson *et al.* 2004; Benton *et al.* 2006). Orco null flies are largely anosmic, though some chemosensation remains due to the presence of ionotropic receptors and gustatory receptors, which do not require Orco to function (Silbering *et al.* 2011; Benton *et al.* 2009; Kwon *et al.* 2007; W. D. Jones *et al.* 2007). The Orco promoter is consequently a convenient device for the expression of transgenes in most OSNs.

The olfactory organs, the antenna and maxillary palp, contain OSNs dendrites within structures called sensilla. ORs and Orco are embedded in the dendritic membrane. OSN axons project to the antennal lobes in the brain of the animal. Each antennal lobe consists of ∼50 globular synaptic sites called glomeruli. All OSNs on the periphery that expresses the same OR converge onto their own unique glomerulus. For example, all OSNs expressing Or22a will send axons to the DM2 glomerulus in the antennal lobe while all OSNs expressing Or82a will send axons to the VA6 glomerulus (Figure 1). The stereotyped organization of OSNs and their projections is known as the olfactory sensory map (Vosshall, Wong, and Axel 2000; Fishilevich and Vosshall 2005; Couto, Alenius, and Dickson 2005; Stocker *et al.* 1990). The regularity of this map is a key feature that makes *Drosophila* olfaction such a useful model, as any aberration to the typical pattern will be apparent. The apparent simplicity of the map (Figure 1), however, obscures mechanistic complexities that are yet to be discovered, in part because necessary tools remain unavailable.

**Figure 1:**
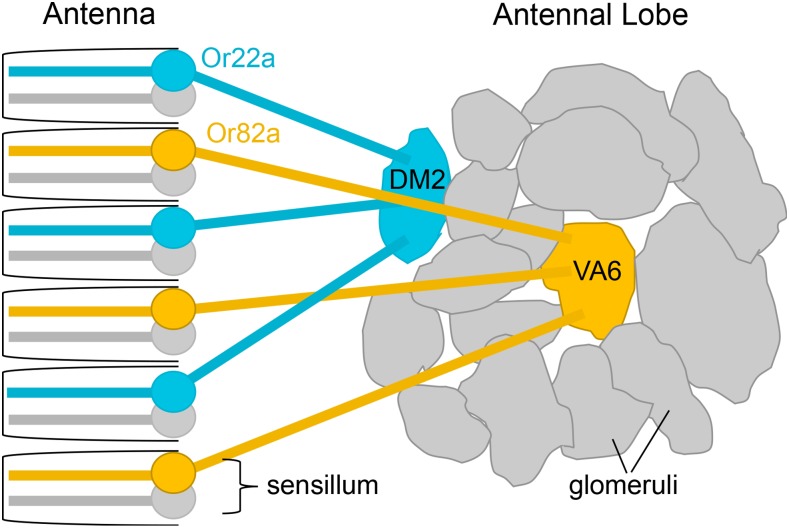
Olfactory Sensory Map. Each neuron in the olfactory system expresses one type of odorant receptor (OR). Or22a (teal) and Or82a (gold) are used here as examples. Neurons usually exist in pairs or groups in sensilla within the olfactory organs—antenna or maxillary palp. Neurons expressing the same OR are distributed throughout the periphery, but project their axons onto the same glomerulus in the antennal lobe of the brain. For example, all Or22a-expressing neurons synapse onto the DM2 glomerulus while all Or82a-expressing neurons synapse onto the VA6 glomerulus.

*Drosophila* geneticists have traditionally relied on genetic mutations or deletions to understand how complex biological systems normally work. Most alleles are recessive, so homozygotes must be bred over multiple generations. Achieving homozygosity of a mutation while also adding transgenes to the system often requires the creation of recombinant chromosomes produced after multiple generations of crossing and PCR screening. Classical genetic strategies thus limit the number and complexity of combinatorial genotypes that one can achieve. More challenging experimental questions demand more facile and versatile genetic tools.

The GAL4/UAS gene regulation system has become a *defacto* standard in studies of *Drosophila*. GAL4 is a yeast transcription activator that binds to the Upstream Activating Sequence (UAS) and induces expression of downstream genes (Giniger, Varnum, and Ptashne 1985). By driving GAL4 expression from an OR promoter, specific expression of a *UAS-transgene* can be obtained for any OSN subtype. An *OrX-GAL4* line exists for almost every OR. This collection of GAL4 lines is a powerful toolbox since different *UAS-transgenes* can be introduced into a line via conventional mating. For example, human α-synuclein has been expressed in OSNs to model human Parkinson’s disease (A. Y. Chen *et al.* 2014). Alternatively, protein expression levels can be knocked down using any specified *UAS-RNAi* transgene.

A variety of existing compatible effectors can be used study different aspects of neuronal communication. The *UAS-Kir2.1* effector is used as an example in experiments described below. This inward rectifier potassium channel electrically inactivates the neurons that express it (Hodge 2009; Baines *et al.* 2001; Johns *et al.* 1999). Similarly, *shibire^ts^* or tetanus toxin can be used to silence synaptic communications (van der Bliek and Meyerowitz 1991; kitamoto 2002; Kitamoto 2001; M. S. Chen *et al.* 1991; Sweeney *et al.* 1995; Baines *et al.* 1999), *reaper/grim/hid* genes can be used to physically kill neurons by inducing their own apoptotic pathways (Song and Steller 1999; Abrams 1999), or ricin toxin can be expressed ectopically to kill neurons. Conversely, neurons can be selectively activated with *trp1a* or a variety of other channelrhodopsin transgenes (Boyden 2011; Pulver *et al.* 2009).

If GAL4 is a standard on-switch for nearly any desired transgene, GAL80 is the logical off-switch. GAL80 binds the GAL4 transcriptional activation domain, thereby preventing recruitment of RNA polymerase (Ma and Ptashne 1987). GAL80 crosses are much more convenient than classical breeding approaches (Figure 2). In order to have a single functional OSN in an otherwise silent olfactory system, the traditional method uses an Orco null mutation (Larsson *et al.* 2004). In this genetic setup, Orco mutant flies are mostly anosmic, but function is restored to one OSN subset with *Or-GAL4*, *UAS-Orco* transgenes (Olsen, Bhandawat, and Wilson 2007; DasGupta and Waddell 2008; Hoare, McCrohan, and Cobb 2008; Hoare *et al.* 2011; Benton *et al.* 2006; Fishilevich *et al.* 2005) (Figure 2a). An *Orco-GAL4*, *UAS-effector*, *Or-GAL80* method can be used instead (Figure 2b). Kir2.1 is used as an example of an effector (Hodge 2009; Baines *et al.* 2001; Johns *et al.* 1999). Classical breeding strategies (Figure 2a) may look less complicated on paper than GAL80 crosses (Figure 2b) but are actually more time-consuming and limited. The Orco mutation must be homozygous. Since most *Drosophila* transgenes are embedded into the same two chromosomes (2 or 3) recombination and PCR screening may be required to achieve this homozygosity.

**Figure 2:**
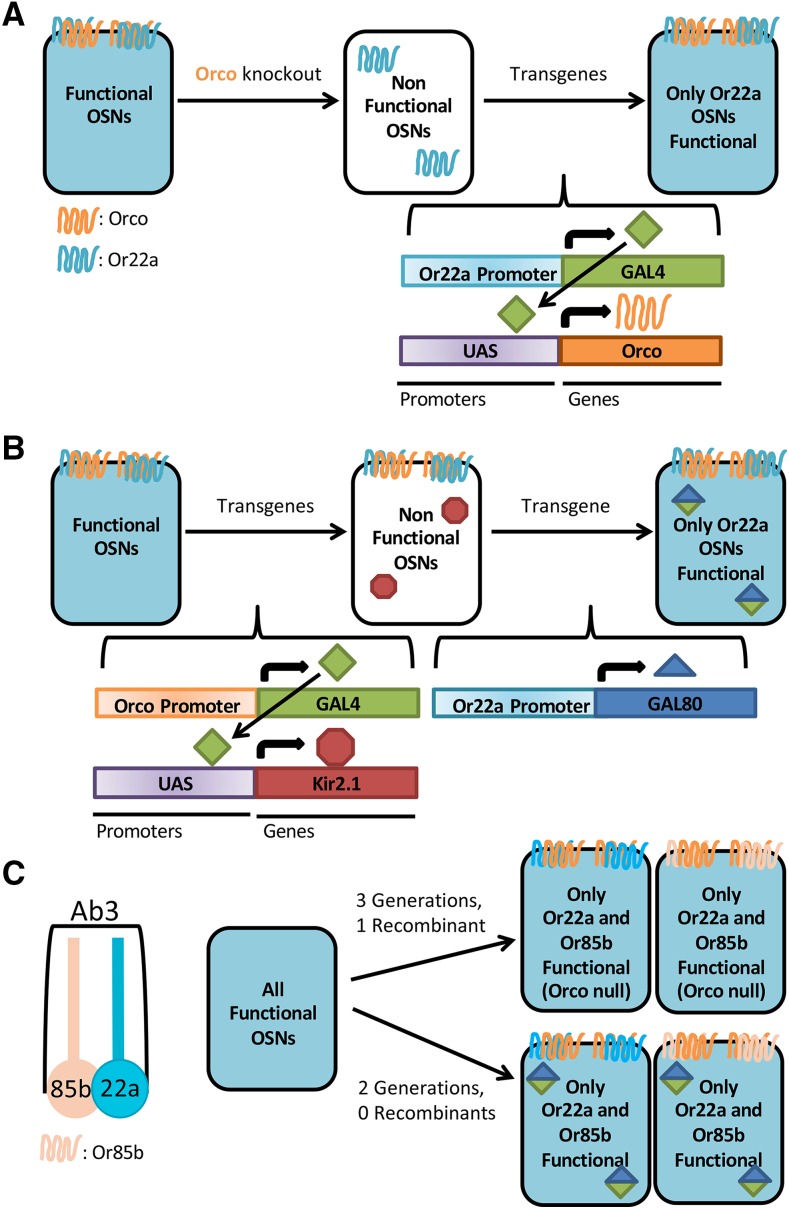
Advantages of using a GAL80 approach over a null mutation. a) Current method with available reagents. In order to examine a single type of Olfactory Sensory Neuron (OSN) without interference from other OSNs, one can use an Orco null mutant. Without Orco, ORs cannot reach the cell membrane or function properly. Orco mutants are mostly anosmic (unable to smell.) A single OR can then be restored using two transgenes, *OrX-GAL4* and *UAS-Orco*. *Or22a-GAL4* is shown here as an example. This fly may require the making and validating of one or more recombinant chromosomes, since the Orco mutation must be homozygous. In more complicated systems, *e.g.*, restoring more than one OSN, multiple recombinants would need to be made and validated at a cost of several months of crossing. b) Using a GAL80. GAL80 is a potent GAL4 inhibitor. All olfactory neurons could be silenced using any number of transgenes in an *Orco-GAL4*, *UAS-effector* (such as *UAS-Kir2.1*) genotype. A single OSN subtype can then be restored using an *OrX-GAL80* (such as *Or22a-GAL80*). This system requires no recombinant creation, and is amenable to the use of various effectors or additional transgenes without requiring recombinant construction. c) Example of a Complex Genotype. If a researcher wants to restore two neurons in an otherwise silent background, using the Orco null approach from 2A would require at least 3 generations and one recombinant creation. Using a GAL80 approach from 2B, one can create the same fly in only 2 generations with no recombination necessary. Or22a (teal) and Or85b (peach) share sensillum Ab3 and are used as an example. Receptor appearance, orientation, and heterodimerization is based on previous designs by Neuhaus *et al.* (2005), Benton *et al.* (2006), Smart *et al.* (2008), and (Smart *et al.* 2008; Neuhaus *et al.* 2005; Benton *et al.* 2006; Benton 2009).

Neurons seldom operate autonomously, but rather groups of neurons coordinate within a circuit to provide an organism with perception and behavior. An investigation of the behavioral impact provided by a limited number of different functional neuronal types would require additional genes. The elaboration of genotypes (Figure 2) to restore pairs or groups of functional OSNs in a nonfunctional background normally requires generations of crosses (followed by PCR screens for desired recombinants). A GAL80 strategy can shorten this process by achieving similar results in only one or two generations with no necessary recombinant creation. Furthermore, a GAL80 strategy takes advantage of the interchangeable variety of existing *UAS-transgene* lines.

For example, Or22a and Or85b are co-expressed in the same sensillum. It is understood that the activity of one neuron in the sensillum affects the activity of another (Su *et al.* 2012). (Gao, Clandinin, and Luo 2015) showed that Or22a was sufficient to restore behavioral responses to E2-Hexenal. But, using a similar apparatus to theirs, we were unable to show any odorant restoration to Isoamyl-Acetate (IAA), another odorant for which Or22a shows a strong response. What is the minimum necessary circuit for restoring IAA perception?

We used our GAL80 system to restore both Or85b and Or22a in an otherwise null system. The two neurons together did not restore behavioral responses (data not shown). An investigator could go further, adding back more OR’s that may contribute to IAA sensation, such as Or10a, which also responds strongly to IAA (Hallem and Carlson 2006).

Using the traditional method, restoration of Or22a and Or85b in an Orco null background would require the creation of at least one recombinant and at least 3 generations of crosses (Figure 2C). And what if the researcher wanted to add a third functional neuron? Or instead of looking a behavior, wanted to look at neuroanatomy and needed to express a reporter gene? The required genotype would be prohibitive. Our GAL80 method required no recombination and only 2 generations. This flexible system could be easily adapted to add more functional neurons, an ectopic receptor, a reporter gene, or any number of transgenes. (Though admittedly, adding more GAL80 transgenes will be easier once the GAL80 construct is targeted to additional landing sites).” Here we describe a new collection of *OrX-GAL80* lines designed to complement existing *OrX-GAL4* lines, and demonstrate their potential utility for neuroanatomical studies of the *Drosophila* olfaction model.

## Materials and Methods

### Fly Stocks

Flies were reared on standard cornmeal/molasses food and kept at 25C with a 16 hr on/8hours off light cycle. All lines were obtained from the Indiana University Bloomington Stock Center and the Janelia Research Campus. Any recombinants made were validated with PCR.

#### Stock List:

Or7a-GAL4 #23907
Or7a-GAL4 #23908
Or9a-GAL4 #23918
Or9a-GAL4 #23919
Or10a-GAL4 #9944
Or13a-GAL4 #9946
Or13a-GAL4 #23886
Or19a-Gal4 #24617
Or22a-GAL4 #9951
Or22a-GAL4 #9952
Or22b-GAL4 #23891
Or33c-GAL4 #23893
Or35a-GAL4 #9967
Or42a-GAL4 #9970
Or42b-GAL4 #9971
Or43b-Gal4 #23894
Or46a-GAL4 #23291
Or47a-GAL4 #9981
Or56a-GAL4 #9988
Or59b-GAL4 #23897
Or59c-GAL4 #23899
Or67a-GAL4 #23904
Or67d-GAL4 #9998
Or71a-GAL4 #23121
Or82a-GAL4 #23125
Orco-GAL4 #23292
Orco-GAL4 #26818
Or85a-GAL4 #23133
Or85b-GAL4 #23911
Or85c-GAL4 #23913
Gr21a-GAL4 #24147
Or22a-mcd8::GFP #52620
Gr21a-mcd8::GFP #52619
Orco^2^ #23130
UAS-Orco #23145
UAS-mcd8::GFP #5130
UAS-mcd8::GFP #5137
UAS-Kir2.1 Janelia stock #3015545
UAS-Kir2.1 Janelia stock #3015298
UAS-Kir2.1::eGFP Janelia stock #BS00312
pJFRC19-13xLexAop2-IVS-myr::GFP-p10 (attP8) Janelia stock #1171146
pJFRC59-13xLexAop2-IVS-myr::GFP-p10 (attP40) Janelia stock #3015445

### GAL80 Creation

Primers were designed to capture the entire promoters described by (Couto, Alenius, and Dickson 2005) (see Table S1). Promoters were amplified from genomic DNA using Q5 High Fidelity PCR (NEB #M0491S) and added to entry vectors using the pENTR/D-TOPO system (Invitrogen 2012b). Recombination with the pBP-GAL80Uw-6 (Addgene #26236) destination vector was done using the LR Clonase II system (Invitrogen 2012a). To ensure no mutations, no gaps, and correct orientation, the complete promoters were sequenced in the destination vector using the sequencing primers shown in Table S2. PhiC31 site-directed transgenesis was performed by Genetivision Inc. All GAL80 transgenes were inserted at the attP2 site, because this landing site is known to produce very strong expression (Pfeiffer *et al.* 2010). Since the widely-used GAL4 collection was not created using the PhiC targeted-landing site system, it is not expected that expression at this site will interfere with the GAL4 genes. Furthermore, most Or-GAL4s are available on multiple chromosomes and therefore a researcher also has a choice of which chromosome to try in a crossing scheme. A single Or-LexA line was also created using the Or22a-promoter entry vector and pBPnlsLexA::p65Uw (Addgene #26230).

### Immunohistochemistry

Female adult brains were dissected one day after eclosion in cold S2 Schneider’s Insect Medium (Sigma Aldrich #S0146) and fixed while nutating for 55 min at room temperature in 2mL 2%PFA (Electron Microscopy Sciences #15713) in protein loBind Tubes (Eppendorf #022431102). Brains were washed 4x, 15min per wash while nutating with 2mL PBT buffer (1xPBS, Cellgro #21-040, with 0.5% TritonX-100, Sigma Aldrich #X100). Brains were then blocked with 200µL 5% Goat serum (ThermoFischer. #16210064) in PBT for 90 min while nutating, upright. Block was removed and 200 µL primary antibodies in PBT were added for 4 hr at room temperature and then transferred to 4C for 36-48 hr while nutating, upright. Primary antibodies: mouse α-bruchpilot (Developmental Studies Hybridoma Bank. #nc82-s) at 1:30, rabbit α-GFP at 1:1000 (Thermo Fischer #A11122), or rabbit α-Tom at 1:500 (clontech #632393). Monoclonal antibody nc82 identifies Bruchpilot. Bruchpilot can serve as a general neuropil marker because it is required in synaptic zones (Wagh *et al.* 2006). Larval brains were collected from third instar larvae and fixed in 4% PFA. Primary antibodies: mouse α-neuroglian (Developmental Studies Hybridoma Bank. #BP104) at 1:50 and rabbit α-GFP at 1:500. Brains were washed 4x, 15min per wash while nutating with 2mL PBT. 200µL secondary antibodies in PBT were then added for 4 hr at room temperature and then 3 overnights at 4C while nutating upright. Secondary antibodies: AF568 goat α-mouse (Life Technologies #A11031) at 1:400 and AF488 goat α-rabbit (ThermoFischer #A11034) at 1:800. Tubes were protected from light at all times after secondary antibodies had been added. Brains were washed again 4x, 15min per wash while nutating with 2mL PBT. Then washed with 1xPBS and mounted using Vectashield mounting media (Vector Labs #H-1000). Confocal images were taken with Leica800 microscope.

### Single Sensillum Recordings

SSRs were performed as described in Lin *et al.* (2015) (Lin and Potter 2015). GFP labeled ab1 and ab3 sensilla were identified using a Zeiss AxioExaminer D1 compound microscope with eGFP filter cube (FL Filter Set 38 HE GFP shift free). A glass recording electrode filled with ringers solution (7.5g of NaCl+0.35g of KCl+0.279g of CaCl_2_-2H_2_O in 1L of H_2_O) was inserted into the base of the sensillum. To test ab1 (Gr21a) response, CO_2_ was delivered through a tube ending with a Pasteur pipette that was inserted for 1 sec into a hole in a plastic pipette directed at the antenna. This plastic pipette (Denville Scientific Inc, 10ml pipette) carried a purified continuous air stream (8.3 ml/s) that used a stimulus controller (Syntech) at the time of CO_2_ delivery to correct for the increased air flow. To test ab3 (Or22a) response, 20 µl of E2-Hexenal or Isoamyl acetate (diluted to 1% in mineral oil) was pipetted on a piece of filter paper (1X2 cm) in a Pasteur pipette. The Pasteur pipette was then inserted into the hole of the plastic pipette that carried continuous air stream to the antenna. For odorant delivery, the stimulus controller (Syntech) was used to divert a 1 s pulse of charcoal-filtered air (5 ml/s) into the Pasteur pipette containing the odorant.

Signals were acquired and analyzed using AUTOSPIKE software (USB-IDAC System; Syntech). Spikes were counted in a 500 ms window from 500 ms after CO_2_ delivery and multiplied by 2 to calculate spikes/second. Then, the spikes in 1000ms before CO_2_ delivery were subtracted to calculate the increase in spike rate in response to CO_2_ (Δspikes/second). For each genotype, 6 flies (4-8 days old) were tested, with 1-3 sensilla tested in each fly.

### Data Availability

All created fly stocks are available through Bloomington Stock Center. Plasmids and raw data available upon request. Supplementary material has been uploaded to figshare. Supplemental material available at Figshare: https://doi.org/10.25387/g3.7125530.

## Results

### Design of GAL80 Constructs

The following criteria were used to choose OR promoters for the collection. i) The ORs should be relevant to current research as shown by the number of studies that used it. ii) The ORs should represent a variety of expression patterns (larval or adult, antennae or maxillary palps, sensillary class etc.). iii) Finally, the ORs should reflect a variety of different odorant response profiles. The promoter regions were defined based largely on the work of Couto *et al.*, (2005).

Equimolar expression of GAL4 and GAL80 is not always sufficient to effectively eliminate GAL4 activity so the pBP-GAL80uW-6 vector was used. This vector contains a modified GAL80 sequence, designed to increase the stability and expression of its gene product (Pfeiffer *et al.* 2010). A few *OrX-GAL80*s were already made with this vector and used effectively. (Gao, Clandinin, and Luo 2015) pioneered the technique by creating a limited number of OrX-GAL80s. This work is a logical extension and makes many additional *OrX-GAL80s* available for general use.

### Testing GAL80 Efficacy and Specificity

**Figure 3:**
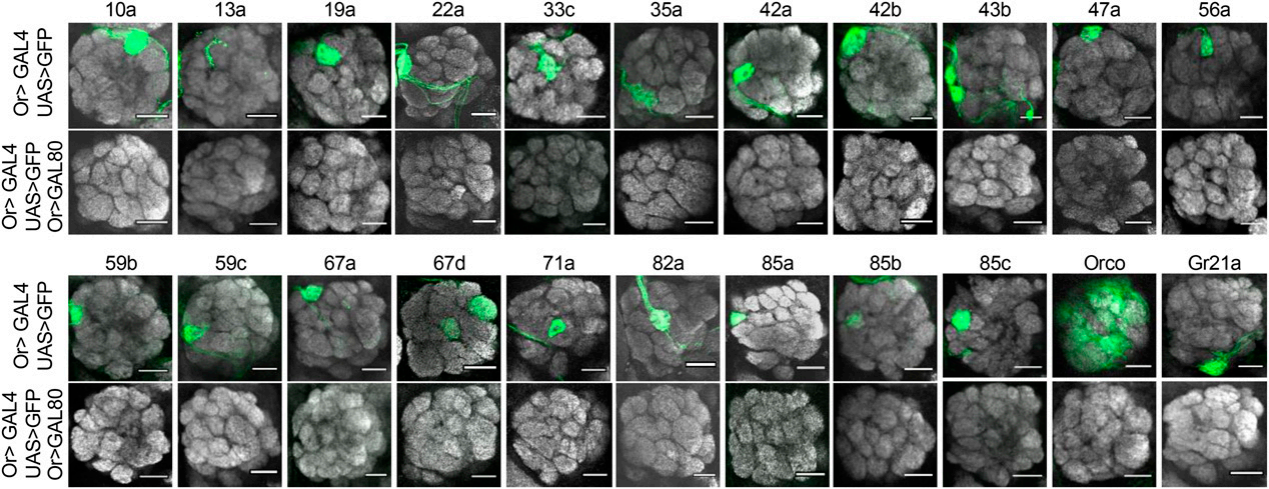
OR-GAL80 reagents eliminate GAL4 activity. All antennal lobes are stained with anti-nc82 (a general neuropil marker, gray) and anti-GFP (green). The orientation of each image is dorsal-up, ventral-down, lateral-right, medial-left. Scale bars indicate 20µm. Each of the brains shown has the genotype *OrX-GAL4*, *UAS-GFP*. The specific receptor promoter is given above each column. The top row in each set shows GFP expression in these lines without GAL80. Notice how each neuron’s target in the antennal lobe glomeruli is expressing GFP. Each bottom row shows the brains containing an additional *Or-GAL80* gene. Note how GAL80 effectively inhibits GAL4 activity, as seen by the elimination of GFP expression. The images are representative of the 5-20 brains examined per genotype. GAL4 inactivation was 100% penetrant in one day old female flies.

GAL80 lines were created for the following odorant receptor promoters: Or7a, Or9a, Or10a, Or13a, Or19a, Or22a, Or22b, Or33c, Or35a, Or42a, Or42b, Or43b, Or47a, Or56a, Or59b, Or59c, Or67a, Or67d, Or71a, Or82a, Orco, Or85a, Or85b, Or85c, and Gr21a. To examine GAL4 subtraction *in vivo*, *OrX-GAL80* flies were crossed to flies with the genotype *OrX-GAL4*, *UAS-mcd8*::*GFP*. OSNs expressing the same OR can be identified from their specific glomerulus in the antennal lobe (Figure 1). *OrX-GAL4*, *UAS-mcd8*::*GFP* flies show robust expression of the membrane-bound GFP reporter gene in their respective glomeruli. However, when *OrX-GAL80* is added to the genotype, GFP expression is entirely absent, indicating a robust antagonism of GAL4 activity (Figure 3). The efficacy of *Or9a-GAL80* and *Or22b-GAL80* could not be determined because their *Or-GAL4*, *UAS-mcd8*::*GFP* controls did not show robust or reliable GFP signaling in the first place. The created *Or7a-GAL80* line was not effective at subtracting GFP signal. Though these lines are not included in Figure 3, they will still be available in the Bloomington Stock Center. Several of the GAL80 lines also have expression in larvae. GAL4 subtraction was examined in larval brains using the *UAS-mcd8*::*GFP* reporter gene. In larvae, GAL80 reduced but did not eliminate GAL4 activity (Figure S1a). Suppression may not be as strong in larvae because the GAL4 and GAL80 genes were under the control of the same promoter. It could require extra time for GAL80 to accumulate to a level that would efficiently inhibit GAL4 activity.

The *OrX-GAL80* lines were checked to ensure they would not have aberrant expression in untargeted OSN subtypes. The pBP-GAL80uW-6 vector contains a Drosophila Synthetic Core Promoter (DSCP). DSCP is an effective means of using enhancer elements to drive strong expression (Pfeiffer *et al.* 2008), but it could also cause the GAL80s to have nonspecific or leaky expression. Therefore, a version of pBP-GAL80uW-6 was cloned with the DSCP removed. However, when the DSCP was absent, GAL80 expression was insufficient to subtract GAL4 activity (Figure S1b). A few lines were tested to see if DSCP causes nonspecific GAL80 expression. For these lines, an *OrY-GAL80* did not impede GAL4 activity of an *OrX-GAL4* neuron (Figure S1c). Due to the uneven expression in an *Orco-GAL4*, *UAS-GFP* line, it could not be determined if each OrX-GAL80 subtracts GAL4 from only one glomerulus in an otherwise fully-labeled brain, but results shown in Figure S1c give reasonable confidence that the GAL80s do not have widespread nonspecific expression. It can also be noted that the GAL80 subtraction does not interfere with reporter gene expression in a genetic system that does not use GAL4. When *Or22a-GAL80* is used in conjunction with *Or22a-mcd8*::*GFP*, containing no GAL4/UAS intermediary, the GFP is still expressed (Figure S1d). These images, showing subtraction of reporter gene expression, confirm that GAL4 activity is suppressed anatomically by the GAL80 lines.

**Figure 4:**
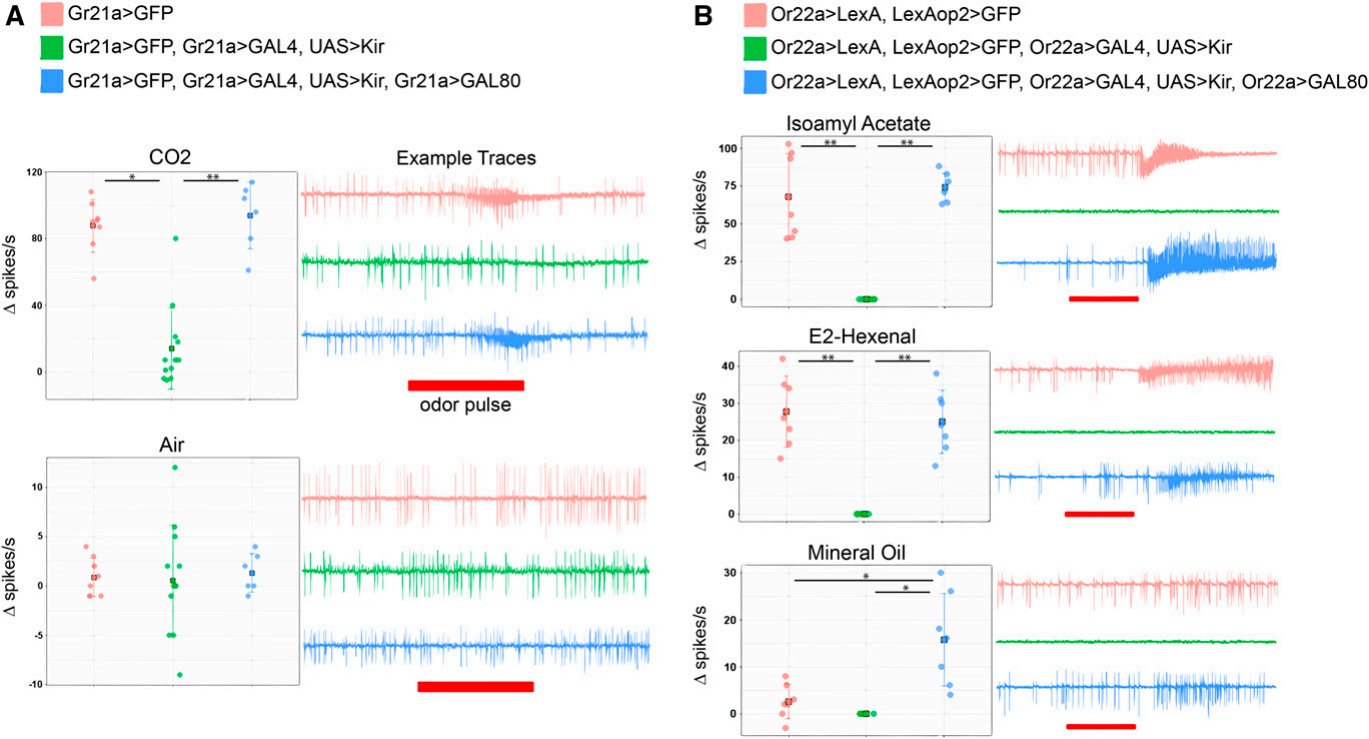
Olfactory neuron responses toward odors in Single Sensillum Recordings (SSR). In box plots on the left, each circle shows response in an individual sensillum, and filled squares indicate the means. On the right of each plot, example SSR traces are shown for each genotype. (* indicates 0.01 > *P* > 0.005, ** indicates *P* < 0.001) a) Ab1C SSR responses. Ab1C neurons are visualized using the *Gr21a-GFP* gene. Top: Sensilla respond strongly to CO_2_, and adding *Kir2.1* reduces response to CO_2._ Response is restored when *Gr21a-GAL80* is added. Bottom: Air was used as a control for CO_2_ experiments. Air does not cause an odor-evoked neuronal response, and adding the *Kir2.1* or *GAL80* genes does not affect the spontaneous signaling responses. b) Ab3 SSR responses. Ab3 neurons are visualized using the *Or22a-LexA and LexAop2-GFP* genes. Sensilla respond to Isoamyl Acetate and to E2-Hexenal. Adding *Kir2.1* eliminates both odor-evoked and spontaneous activity in these neurons_._ Spontaneous and odor-evoked activity is restored when *Or22a-GAL80* is added. Odorants were diluted in mineral oil and neurons from the GAL80 restorative genotype did show low-level responses to mineral oil alone (bottom).

To confirm GAL4 was suppressed physiologically by the GAL80s, Single Sensillum Recordings (SSRs) were used to assay electrical activity of OSNs. *Gr21a-mcd8*::*GFP* was used to identify sensilla of interest without interfering with the GAL4/UAS/GAL80 system. Gr21a neurons are housed in ab1 sensilla. Carbon Dioxide exposure causes a robust response in Gr21a ab1C neurons (Hallem and Carlson 2006; Hallem, Ho, and Carlson 2004; Fishilevich and Vosshall 2005; deBruyne:2001bs W. D. Jones *et al.* 2007; Kwon *et al.* 2007; de Bruyne, Foster, and Carlson 2001). When *Gr21a-mcd8*::*GFP* flies were exposed to CO_2_, their ab1C sensillar neurons showed robust responses (mean Δspikes/s = 88, N = 8 sensilla). Kir2.1-containing neurons are expected to show little to no spontaneous firing (Olsen, Bhandawat, and Wilson 2007; Hoare, McCrohan, and Cobb 2008). Adding Kir2.1 to Gr21a neurons (genotype *Gr21a-mcd8*::*GFP*, *Gr21a-GAL4*, *UAS-Kir2.1)* greatly reduced spiking responses to CO_2_ (mean Δspikes/s = 14, N = 12 sensilla, *P* = 0.01). When Gr21a-GAL80 was added (genotype *Gr21a-mcd8*::*GFP*, *Gr21a-GAL4*, *UAS-Kir2.1*, *Gr21a-GAL80)*, responses to CO_2_ were restored (mean Δspikes/s = 94, N = 6 sensilla, *P* < 0.001. No significant difference from genotype *Gr21a-GFP*, *P* = 0.26) (Figure 4a).

To make sure the system also worked for neurons expressing an OR protein (in additional to a GR), SSR was also done with ab3 sensilla. Ab3 houses Or22a-expressing neurons. This receptor is known to be activated by a diverse set of odorants, including Isoamyl acetate and E2-hexenal (Hallem and Carlson 2006; Hallem, Ho, and Carlson 2004; Fishilevich and Vosshall 2005; deBruyne:2001bs W. D. Jones *et al.* 2007; Kwon *et al.* 2007; de Bruyne, Foster, and Carlson 2001). A transgene was necessary to visualize the neurons without interfering with the GAL4/UAS/GAL80 system, but *Or22a-mcd8*::*GFP* was insufficiently bright to identify sensilla for SSR. A new *Or22a-LexA* transgenic animal was therefore created using the same promoter that was used to create the *Or22a-GAL80* gene (this line is also available through Bloomington). When crossed to a *LexAop2-mcd8*::*GFP* line, the ab3 sensilla showed bright fluorescence. When *Or22a-LexA*, *LexAop2-mcd8*::*GFP* flies were exposed to Isoamyl acetate or to E2-hexenal, their sensillar neurons showed robust responses (mean Δspikes/s = 67.86 and 27.71, N = 7 and 7 sensilla, respectively). Unlike the Gr21a neurons, Kir2.1 expression in the Or22a neurons effectively eliminated both spontaneous and odor-evoked activity. (mean Δspikes/s = 0, N = 7 sensilla, *P* < 0.001 for both odorants). Both activities could be restored with the addition of the *Or22a-GAL80* gene (Isoamyl acetate: mean Δspikes/s =74.29, N = 7 sensilla, *P* < 0.001; E2-Hexenal: mean Δspikes/s =25, N = 7 sensilla, *P* < 0.001). Neurons showed some low-level responses to mineral oil alone, the solvent used for the odorants (Figure 4b). Only genotype 3 *Or22a-LexA*, *LexAop2-mcd8*::*GFP*, *Or22a-GAL4*, *UAS-Kir2.1*, *Or22a-GAL80* showed significantly higher responses to mineral oil than genotypes *Or22a-LexA*, *LexAop2-mcd8*::*GFP* (*P* = 0.01) and *Or22a-LexA*, *LexAop2-mcd8*::*GFP*, *Or22a-GAL4*, *UAS-Kir2.1* (*P* = 0.005), but the latter two genotypes showed no significant response to mineral oil alone. The results in Figure 4 confirm that GAL80 functions effectively to prevent GAL4-induced activity in OSNs.

## Discussion

The collection of GAL80 lines subtracts GAL4 activity efficiently and specifically in OSNs. In anatomical studies, reporter gene expression from the GAL4/UAS system is suppressed. The tested neuronal subtypes, which were silenced with Kir2.1, have normal firing capacity restored when GAL4 is antagonized using the GAL80 lines.

In behavioral assays, using a GAL80 transgene will be more flexible than mutant lines and less cumbersome than crafting the required recombinants as the complexity of the genotype increases. Though in some special circumstances, olfactory sensory neurons have been shown to produce behaviors autonomously, this is not a widely applicable principle, and further investigation upon this principle requires better tools. For example, Fishilevich *et al.* (2005) used larvae in their study to restore aversion with a single functional OSN subtype, but the larval olfactory system may be fundamentally different from adults in this respect., (Bhandawat *et al.* 2010) also showed that single glomerular activity is sufficient to invoke a behavioral response, but that study was done using an intact and fully functional olfactory background, so some neuronal cooperation may still have occurred. (DasGupta and Waddell 2008) provided evidence that a single functional OSN subtype is sufficient to learn odor discrimination, and Gao *et al.* (2015) gave convincing evidence of aversive restoration in adults with only one functional OSN.

However, the extent to which the restoration of single-OSN behavior depends on the odorant and receptor used is still unknown. Only a small subset of receptors have been tested. The current models of odor coding by the olfactory system predict that a coordinated effort of many OSNs is usually required to produce a behavioral output. Paired neurons in sensilla can affect the firing dynamics of their neighbors in the periphery (Dobritsa *et al.* 2003; Su *et al.* 2012; Kazama and Wilson 2009), and downstream neurons such as interneurons and projection neurons may rely on synchronized input from multiple OSN types (Chou *et al.* 2010; Wilson 2011; Yaksi and Wilson 2010; Hong and Wilson 2015; Kazama, Yaksi, and Wilson 2011; Olsen, Bhandawat, and Wilson 2007; Ng *et al.* 2002; Acebes *et al.* 2011). GAL80 tools open more possibilities to combinatorially activate subsets of neurons. The hope is that additional researchers will use the reagents and validate them in their own assays.

Researchers encounter a significant technical obstacle to the understanding of olfactory function if they need to create genotypes with small groups of interacting neurons in isolation. The tools presented here facilitate the activation or deactivation of combinations of particular neurons, thereby overcoming this obstacle. The lines are available to order through Bloomington Stock Center.
